# Improving the provision of pregnancy care for Aboriginal and Torres Strait Islander women: a continuous quality improvement initiative

**DOI:** 10.1186/s12884-016-0892-1

**Published:** 2016-05-24

**Authors:** Melanie E. Gibson-Helm, Alice R. Rumbold, Helena J. Teede, Sanjeeva Ranasinha, Ross S. Bailie, Jacqueline A. Boyle

**Affiliations:** Monash Centre for Health Research and Implementation, School of Public Health and Preventive Medicine, Monash University, Melbourne, VIC Australia; The Robinson Research Institute, The University of Adelaide, North Adelaide, South Australia Australia; Menzies School of Health Research, Charles Darwin University, Brisbane, QLD Australia; Diabetes and Vascular Medicine, Monash Health, Melbourne, VIC Australia

**Keywords:** Indigenous health services, Australia, Quality improvement, Pregnancy, Primary health care, Maternal health

## Abstract

**Background:**

Australian Aboriginal and Torres Strait Islander (Indigenous) women are at greater risk of adverse pregnancy outcomes than non-Indigenous women. Pregnancy care has a key role in identifying and addressing modifiable risk factors that contribute to adverse outcomes. We investigated whether participation in a continuous quality improvement (CQI) initiative was associated with increases in provision of recommended pregnancy care by primary health care centers (PHCs) in predominantly Indigenous communities, and whether provision of care was associated with organizational systems or characteristics.

**Methods:**

Longitudinal analysis of 2220 pregnancy care records from 50 PHCs involved in up to four cycles of CQI in Australia between 2007 and 2012. Linear and logistic regression analyses investigated associations between documented provision of pregnancy care and each CQI cycle, and self-ratings of organizational systems. Main outcome measures included screening and counselling for lifestyle-related risk factors.

**Results:**

Women attending PHCs after ≥1 CQI cycles were more likely to receive each pregnancy care measure than women attending before PHCs had completed one cycle e.g. screening for cigarette use: baseline = 73 % (reference), cycle one = 90 % [odds ratio (OR):3.0, 95 % confidence interval (CI):2.2-4.1], two = 91 % (OR:5.1, 95 % CI:3.3-7.8), three = 93 % (OR:6.3, 95 % CI:3.1-13), four = 95 % (OR:11, 95 % CI:4.3-29). Greater self-ratings of overall organizational systems were significantly associated with greater screening for alcohol use (β = 6.8, 95 % CI:0.25-13), nutrition counselling (β = 8.3, 95 % CI:3.1-13), and folate prescription (β = 7.9, 95 % CI:2.6-13).

**Conclusion:**

Participation in a CQI initiative by PHCs in Indigenous communities is associated with greater provision of pregnancy care regarding lifestyle-related risk factors. More broadly, these findings support incorporation of CQI activities addressing systems level issues into primary care settings to improve the quality of pregnancy care.

## Background

Large disparities in pregnancy outcomes exist between Aboriginal and Torres Strait Islander (Indigenous) women and non-Indigenous women in Australia. Low birth weight, preterm birth, perinatal death and neural tube defects are more common in babies of Indigenous women [1–3] and lifestyle-related risk factors such as smoking, alcohol use, low peri-conception folate use and poor nutrition are also more common among Indigenous women [2–6]. These risk factors are associated with socio-economic disadvantage with education, employment, income and housing security generally lower among Indigenous populations relative to other Australian groups [7]. Indigenous women can experience barriers to accessing adequate pregnancy care such as transport, financial or language issues, and availability of culturally appropriate care [8, 9].

Approximately 4 % of all women giving birth in Australia identify as being Indigenous although this varies considerably across jurisdictions from 3 % in New South Wales to 36 % in the Northern Territory [2]. Population size also differs across jurisdictions; the largest absolute number of Indigenous women giving birth is in Queensland (3,646) and the smallest in the Northern Territory (1,414). Across Australia a minority of women (3 %) giving birth live in geographically remote areas although this also varies across jurisdictions (ranging from 0.7 % in New South Wales to 48 % in the Northen Territory) and by Indigenous status (27 % of Indigenous women compared to 2 % of non-Indigenous women) [2]. The type of pregnancy care available in Australia often depends on service availability, the woman’s preferences and her risk profile [8]. It can consist of primary and/or hospital care and often involves a range of health professionals [8]. In remote locations, pregnancy care is usually provided by primary health care centers (PHCs), with women transferring to regional hospitals for the birth. PHCs can either be governed by the jurisdiction’s government or be one of over 150 community-controlled health services initiated and operated by the local Indigenous community to deliver holistic, comprehensive, and culturally appropriate health care [10].

Pregnancy care has a key role in identifying and addressing risks of adverse health outcomes for mother and baby, including lifestyle factors. It provides opportunities for shared decision making and the promotion of good health [8]. However, effective long-term strategies across the diverse range of settings in Australia are needed to ensure Indigenous women receive recommended pregnancy care [11].

One potential strategy is Continuous Quality Improvement (CQI) which is a management approach or set of principles that aims to constantly increase the efficiency and effectiveness of organizational systems to better meet the needs and expectations of patients and other stakeholders [12, 13]. These principles include a positive focus on better functioning of organizational systems rather than isolated issues or personal blame, and organizational-wide involvement to foster ownership and build quality improvement capacity [12, 14]. Implementation of CQI consists of ongoing cycles of systematic data collection, using the data for benchmarking and setting goals related to organizational processes and structures, developing best practice improvement strategies, strategy implementation, and subsequent evaluation using iterative data collection [14]. CQI is an approach that has been used successfully in Indigenous primary health care in Australia relating to chronic disease prevention, diabetes and rheumatic heart disease [15–17].

Previous research has identified areas for improvement in pregnancy care by PHCs in Indigenous communities. Nearly 50 % of women attended in the first trimester, providing important opportunities for preventive pregnancy care [18]. Women also attended PHCs regularly during their pregnancy yet did not necessarily receive all components of recommended pregnancy care, especially relating to lifestyle factors [18]. Building on this, we investigated whether provision of care related to these risk factors increased after PHC participation in a large-scale CQI initiative implemented across multiple settings. We also explored whether improvements were associated with organizational systems or characteristics.

## Methods

### Study design and setting

The Audit and Best Practice for Chronic Disease (ABCD) National Research Partnership is a research network that links multiple PHCs in CQI to improve provision of health care in Indigenous communities. By including many PHCs across diverse settings, it is designed to address systems-level issues that commonly affect health care provision. The study protocol and planning for the ABCD National Research Partnership have been described in detail previously [18–20]. One of the main partners of the ABCD Partnership is One21seventy, the National Centre for Quality Improvement in Indigenous Health, which provides evidence-based CQI tools, training, ongoing support and facilitation [21]. Specific tools are designed for different areas of health care e.g. pregnancy care, chronic disease, child health etc. PHCs can choose to focus on one or multiple areas of care at the same time, or to focus on different areas in different years. The ABCD National Research Partnership accesses data collected in One21seventy activities from PHCs that also consented to participate in the research partnership. In summary, each PHC chooses their own care priorities and strategies for improvement at the local level but the underlying approach and principles, the process and data collection tools are standardised, enabling PHCs, One21Seventy, policy makers and researchers to collaborate in large-scale systems research.

### Intervention: CQI cycles

At the beginning of a CQI cycle, two types of data are collected (tools described below). A participatory “systems assessment” identifies strengths and weaknesses and facilitates staff to come to an agreement about how well their organisational systems are currently functioning. Audits of patient health records provide information about the frequency of current provision of recommended care. Together, these data are used by staff to identify priorities for improvement in organizational systems in order to improve service delivery. Staff then develop strategies to achieve these goals. Repeated systems assessments and clinical audits in subsequent years then assess the effectiveness of these strategies in improving care, and identify the next priorities for improvement. These processes form one CQI cycle and PHCs are encouraged to conduct one cycle at least annually, to support long-term CQI implementation.

This article reports longitudinal analysis of data from 50 PHCs conducting maternal health audits for up to four CQI cycles in Australia between 2007 and 2012.

### Tools

#### Systems assessment

A structured assessment of PHC systems was conducted by PHC staff and a trained external CQI facilitator in a consensus process using the Systems Assessment Tool (SAT) [22]. The SAT is based on the Chronic Care Model and the Assessment of Chronic Illness Care scale [23] and was designed to support CQI initiatives in primary health care. It produces an overall mean score (0–11) for the state of development of PHC systems and five subscale scores: (i) delivery system design (e.g. continuity of care), (ii) information systems and decision support (e.g. evidence-based guidelines), (iii) self-management support (e.g. providing appropriate education and behavior change interventions), (iv) links with community and other services, and (v) organizational influence and integration (e.g. organizational culture).

#### Maternal health audits

Recorded delivery of recommended pregnancy care was assessed by auditing a sample of health records from participating PHCs [18]. Audits were conducted by trained auditors (PHC staff, staff from other PHCs or CQI facilitators) supported by a standard protocol and regional CQI facilitators. Audit tool items were derived from best practice guidelines, policy and research reports, and stakeholder consultation [18]. Records of women with an infant aged between 2–14 months and who resided in the community for at least six months of that pregnancy were eligible. The audit protocol provided sample size guidance to help ensure the sample reflected the general population of women attending the PHC.

### Key outcome measures

This study focused on documented measures of recommended pregnancy care regarding modifiable, lifestyle-related risk factors that contribute to adverse health outcomes. Measures were selected from the maternal health audit: screening for cigarette use (non-smoker or any cigarette use during the pregnancy), screening for alcohol use (no alcohol consumption or any consumption during the pregnancy), provision of cigarette cessation advice and brief alcohol counselling (of those screened and who reported use), folate prescription prior to 20 weeks gestation, and counselling about nutrition, physical activity and food security. The Australian Clinical Practice Guidelines for Antenatal Care recommend discussing these risk factors with all women during their first pregnancy care visit [8].

### Statistical analysis

Statistical analyses were performed using Stata software version 12.1 (StataCorp, College Station, Texas, USA) and a two-sided p-value of <0.05 was considered statistically significant. Descriptive statistics (Table 1) are presented as count and proportion for categorical data and median and interquartile range for continuous data. Two types of regression analyses were performed; the first used health record (individual) level data (Table 2) and the second used PHC level data (Table 3).

**Table 1:** Characteristics of primary health centers (PHCs) participating in a CQI initiative according to the total number of CQI cycles completed by each PHC, characteristics of PHCs involved in each CQI cycle and women whose health records were audited as part of each CQI cycle

	Total number of CQI cycles completed* by each PHC				
Characteristics of PHCs	1	2	3	4	
Number of PHCs *n* = 50	22	20	2	6	
Community governed (%)	1 (5.0)	9 (45)	2 (100)	6 (100)	
Population size over 1000 (%)	12 (55)	9 (45)	1 (50)	3 (50)	
Located in remote community (%)	15 (68)	17 (85)	2 (100)	4 (67)	
State: New South Wales (%)	0	2 (10)	0	4 (67)	
Northern Territory	3 (14)	11 (55)	1 (50)	1 (17)	
South Australia	1 (4.6)	0	0	0	
Queensland	14 (64)	7 (35)	0	0	
Western Australia	4 (18)	0	1 (50)	1 (17)	
Mean baseline provision	1	2	3	4	
Screened for cigarette use %	79	65	70	82	
Cigarette cessation advice %	61	56	56	16	
Screened for alcohol use %	71	57	61	59	
Brief alcohol counselling %	52	61	32	17	
Nutrition counselling %	43	48	17	40	
Food security counselling %	3.8	7.0	1.7	0.56	
Physical activity counselling %	24	26	1.7	6.8	
Folate prescription < 20 weeks %	34	30	14	4.5	
	Longitudinal Characteristics				
Characteristics of PHCs	Baseline	End of cycle 1	End of cycle 2	End of cycle 3	End of cycle 4
Number of PHCs *n* = 50	50	50	28	8	6
Community governed (%)	18 (36)	18 (36)	17 (61)	8 (100)	6 (100)
Population size over 1000 (%)	25 (50)	25 (50)	13 (46)	4 (50)	3 (50)
Located in remote community (%)	38 (76)	38 (76)	23 (82)	6 (75)	4 (67)
State: New South Wales (%)	6 (12)	6 (12)	6 (21)	4 (50)	4 (67)
Northern Territory	16 (32)	16 (32)	13 (46)	2 (25)	1 (17)
South Australia	1 (2.0)	1 (2.0)	0	0	0
Queensland	21 (42)	21 (42)	7 (25)	0	0
Western Australia	6 (12)	6 (12)	2 (7.1)	2 (25)	1 (17)
Characteristics of women	Baseline	end of cycle 1	end of cycle 2	end of cycle 3	end of cycle 4
Number of women *n* = 2220	829	758	388	135	110
Median age at delivery, years (IQR) *n* = 2214	24 (20–30) *n* = 824	25 (20–29) *n* = 757	24 (21–29) *n* = 388	25 (21–30) *n* = 135	24 (20–30) *n* = 110
Aboriginal and/or Torres Strait Islander (%) *n* = 2074	670/790 (85)	596/695 (86)	318/346 (92)	119/133 (89)	93/110 (85)
Median number of pregnancy care visits (IQR) *n* = 2220	7/829 (4–10)	8/758 (5–11)	7/388 (4–9)	7/135 (4–9)	8/110 (6–10)
Attendance <13 weeks (%) *n* = 2219	387/829 (47)	418/757 (55)	202/388 (52)	65/135 (48)	74/110 (67)

*CQI* continuous quality improvement, *CI* confidence interval, *IQR* interquartile range *One completed cycle involves auditing health records (baseline), identifying priorities for improvement, developing and implementing strategies to achieve these goals, and a repeated audit to assess improvement (end of cycle 1)

**Table 2:** Longitudinal recorded provision of pregnancy care measures regarding lifestyle-related risk factors, after each CQI cycle and compared to baseline

	CQI cycle					
Number of women receiving each outcome measure	Baseline *n* = 829	End of cycle 1 *n* = 758	End of cycle 2 *n* = 388	End of cycle 3 *n* = 135	End of cycle 4 *n* = 110	*p*-value for trend
Screened for cigarette use (%)	603 (73)	679 (90)	352 (91)	125 (93)	105 (95)	
OR (95 % CI)	ref	3.0* (2.2 to 4.1)	5.1* (3.3 to 7.8)	6.3* (3.1 to 13)	11* (4.3 to 29)	n.s.
OR (95 % CI) adjusted for mean baseline provision^a^	ref	3.0* (2.2 to 4.1)	5.2* (3.3 to 8.0)	6.3* (3.1 to 13)	11* (4.3 to 29)	n.s.
Cigarette cessation advice (%)	185/357 (52)	280/403 (69)	161/238 (68)	63/92 (68)	49/67 (73)	
OR (95 % CI)	ref	2.0* (1.5 to 2.8)	2.6* (1.7 to 4.0)	5.8* (3.1 to 11)	11* (5.2 to 22)	<0.001
OR (95 % CI) adjusted for mean baseline provision^a^	ref	2.1* (1.5 to 2.9)	2.7* (1.8 to 4.2)	7.0* (3.7 to 13)	14* (6.5 to 28)	<0.001
Screened for alcohol use (%)	539 (65)	633 (84)	328 (85)	111 (82)	91 (83)	
OR (95 % CI)	ref	2.6* (2.0 to 3.4)	3.7* (2.6 to 5.4)	3.0* (1.7 to 5.1)	3.8* (2.1 to 6.9)	n.s.
OR (95 % CI) adjusted for mean baseline provision^a^	ref	2.6* (2.0 to 3.5)	3.9* (2.7 to 5.7)	3.0* (1.8 to 5.2)	3.9* (2.2 to 7.1)	n.s.
Brief alcohol counselling (%)	87/172 (51)	135/191 (71)	42/63 (67)	23/40 (58)	17/24 (71)	
OR (95 % CI)	ref	2.8* (1.7 to 4.6)	2.0 (1.0 to 4.0)	2.2 (0.9 to 5.2)	4.5* (1.6 to 13)	n.s.
OR (95 % CI) adjusted for mean baseline provision^a^	ref	2.8 (1.7 to 4.5)	2.0* (1.0 to 4.1)	3.1* (1.3 to 7.2)	6.7* (2.3 to 20)	<0.001
Nutrition counselling (%)	337 (41)	436 (58)	227 (59)	68 (50)	71 (65)	
OR (95 % CI)	ref	1.9* (1.5 to 2.4)	2.7* (2.0 to 3.6)	3.5* (2.3 to 5.5)	8.5* (5.2 to 14)	<0.001
OR (95 % CI) adjusted for mean baseline provision^a^	ref	1.9* (1.5 to 2.4)	2.7* (2.0 to 3.6)	3.6* (2.3 to 5.6)	8.6* (5.2 to 14)	<0.001
Food security counselling (%)	40 (4.8)	144 (19)	75 (19)	16 (12)	10 (9.1)	
OR (95 % CI)	ref	4.6* (3.1 to 7.0)	3.2* (1.9 to 5.4)	7.9* (3.5 to 18)	11* (4.2 to 28)	n.s.
OR (95 % CI) adjusted for mean baseline provision^a^	ref	4.6* (3.1 to 7.0)	3.1* (1.9 to 5.2)	8.7* (3.8 to 20)	12* (4.7 to 33)	n.s.
Physical activity counselling (%)	162 (20)	232 (31)	125 (32)	30 (22)	17 (15)	
OR (95 % CI)	ref	1.7* (1.3 to 2.2)	1.9* (1.4 to 2.7)	3.2* (1.8 to 5.6)	3.3* (1.7 to 6.5)	<0.001
OR (95 % CI) adjusted for mean baseline provision^a^	ref	1.7* (1.3 to 2.2)	2.0* (1.4 to 2.8)	3.9* (2.2 to 6.9)	4.0* (2.0 to 7.9)	<0.001
Folate prescription < 20 weeks (%)	234 (28)	329 (43)	143 (37)	57 (42)	37 (34)	
OR (95 % CI)	ref	2.0* (1.6 to 2.5)	2.3* (1.7 to 3.2)	5.3* (3.2 to 8.6)	4.7* (2.8 to 8.2)	n.s.
OR (95 % CI) adjusted for mean baseline provision^a^	ref	2.0* (1.6 to 2.6)	2.4* (1.7 to 3.3)	6.0* (3.7 to 9.9)	5.5* (3.2 to 9.6)	n.s.

*p < 0.05, *CQI* continuous quality improvement, *OR* odds ratio, *CI* confidence interval, *ref* reference group, *n.s*. not significant for trend. Using each health record as the unit of analysis, random effects logistic regression analysis assessed any associations between provision of pregnancy care measures and completion of 1–4 CQI cycles, with adjustment for clustering of health records within PHCs. ^a^adjusted for mean baseline provision per total cycles completed (given in Table 1)

**Table 3:** Associations between average proportions of women receiving a pregnancy care measure (outcome measures) and average Systems Assessment Tool scores (predictors)

Outcome measure	Overall systems	Delivery system design	Information systems and decision support	Self-management support systems	External links	Organizational influence and integration
Screened for cigarette use β coefficient (95 % CI)	2.6 (−3.1 to 8.3)	1.3 (−4.4 to 7.0)	4.0 (−0.87 to 8.9)	2.1 (−1.7 to 5.9)	0.37 (−4.6 to 5.3)	1.3 (−3.9 to 6.4)
Cigarette cessation advice β (95 % CI)	6.8 (−0.18 to 14)	7.1* (0.33 to 14)	2.3 (−4.4 to 9.0)	5.9* (1.5 to 10)	1.2 (−5.2 to 7.6)	7.2* (1.2 to 13)
Screened for alcohol use β (95 % CI)	6.8* (0.25 to 13)	5.2 (−1.5 to 12)	7.6* (2.0 to 13)	5.0* (0.62 to 9.3)	2.5 (−3.5 to 8.5)	4.3 (−1.8 to 10)
Brief alcohol counselling β (95 % CI)	6.3 (−2.9 to 15)	5.4 (−3.5 to 14)	2.5 (−6.3 to 11)	5.3 (−0.62 to 11)	1.6 (−6.3 to 9.6)	6.7 (−1.4 to 15)
Nutrition counselling β (95 % CI)	8.3* (3.1 to 13)	6.6* (1.2 to 12)	7.4* (2.8 to 12)	4.7* (1.0 to 8.4)	4.0 (−1.0 to 9.0)	7.3* (2.8 to 12)
Folate prescription < 20 weeks β (95 % CI)	7.9* (2.6 to 13)	6.1* (0.44 to 12)	6.7* (1.8 to 12)	3.7 (−0.23 to 7.7)	4.9 (−0.01 to 9.9)	8.2* (3.7 to 13)

*p < 0.05, *n* = 27 PHCs except *n* = 26 for cigarette cessation advice and *n* = 25 for brief alcohol counselling. *β* beta coefficient, *CI* confidence interval. Using each PHC as the unit of analysis, univariable linear regression assessed associations between the average proportion of women who received each pregnancy care measure across all cycles and average overall or subscale SAT scores

#### Associations between completion of CQI cycles and pregnancy care measures (individual level, Table 2)

Using each woman’s health record as the subject or unit of analysis, random effects logistic regression analysis assessed associations between completion of each CQI cycle (predictor) and each key outcome measure (components of recommended pregnancy care). Random effects logistic regression allows for repeated measures of each outcome after each CQI cycle (longitudinal analysis) and for adjustment for similarities between health records within each PHC (PHC as the random effect). The reference group was data collected at the first audit, before PHCs commenced maternal health CQI activities. These analyses were also repeated to include mean baseline provision per total number of completed cycles, to control for baseline performance. We also tested for a trend for increases in provision of outcome measures with each additional CQI cycle completed.

#### Associations between SAT scores and pregnancy care measures (PHC level, Table 3)

Using each PHC as the subject or unit of analysis, univariable linear regression assessed associations between:

1) average SAT scores and the average proportion of women who received each outcome measure across all cycles (cross-sectional analysis). This assessed whether the state of development of PHC systems (predictor) was associated with provision of recommended pregnancy care (outcome).

2) total change in SAT scores and the total change (from first to last cycle) in the proportion of women who received each outcome measure. This assessed whether increased development of PHC systems (predictor) was associated with increased provision of recommended pregnancy care (outcome).

#### Associations between PHC characteristics and pregnancy care measures

Pearson chi-square or Fisher exact tests assessed associations between PHC characteristics (predictors: governance, population size, location and state) and provision of recommended pregnancy care (outcome). For each outcome measure, PHCs were divided into tertiles based on the average proportion of women who received the measure across all cycles (cross-sectional analysis). We also examined associations between PHC characteristics and total change in each outcome measure between first and last cycles.

## Results

The study sample comprised 2,220 records from 50 PHCs. Commencement year varied with 19 PHCs beginning to audit maternal health records in 2007, 9 in 2008, 10 in 2009, 6 in 2010 and 6 in 2011. In some years PHCs may focus their CQI activities on other clinical areas; 11 PHCs audited maternal health records in non-consecutive years. Characteristics of PHCs and women attending for pregnancy care are described in Table 1. A range of PHC governance structures, population sizes, locations and states was represented. The majority of women were Indigenous and 47-67 % first attended for pregnancy care in the first trimester.

### Associations between completion of CQI cycles and pregnancy care measures (individual level)

Provision of each outcome measure after each CQI cycle is shown in Table 2. Random effects logistic regression analysis showed women attending after PHCs had completed one or more CQI cycles were more likely to receive all of the outcome measures investigated (Table 2). For example, the odds of women receiving screening for cigarette use after cycles one, two, three or four were 3.0, 5.1, 6.3 and 11 times greater than before PHCs had begun maternal health CQI activities (baseline). However, scope for further improvement remained for most measures. There was evidence of a trend between an increase in total number of CQI cycles and an increase in provision of four of the outcome measures.

**Fig. 1:**
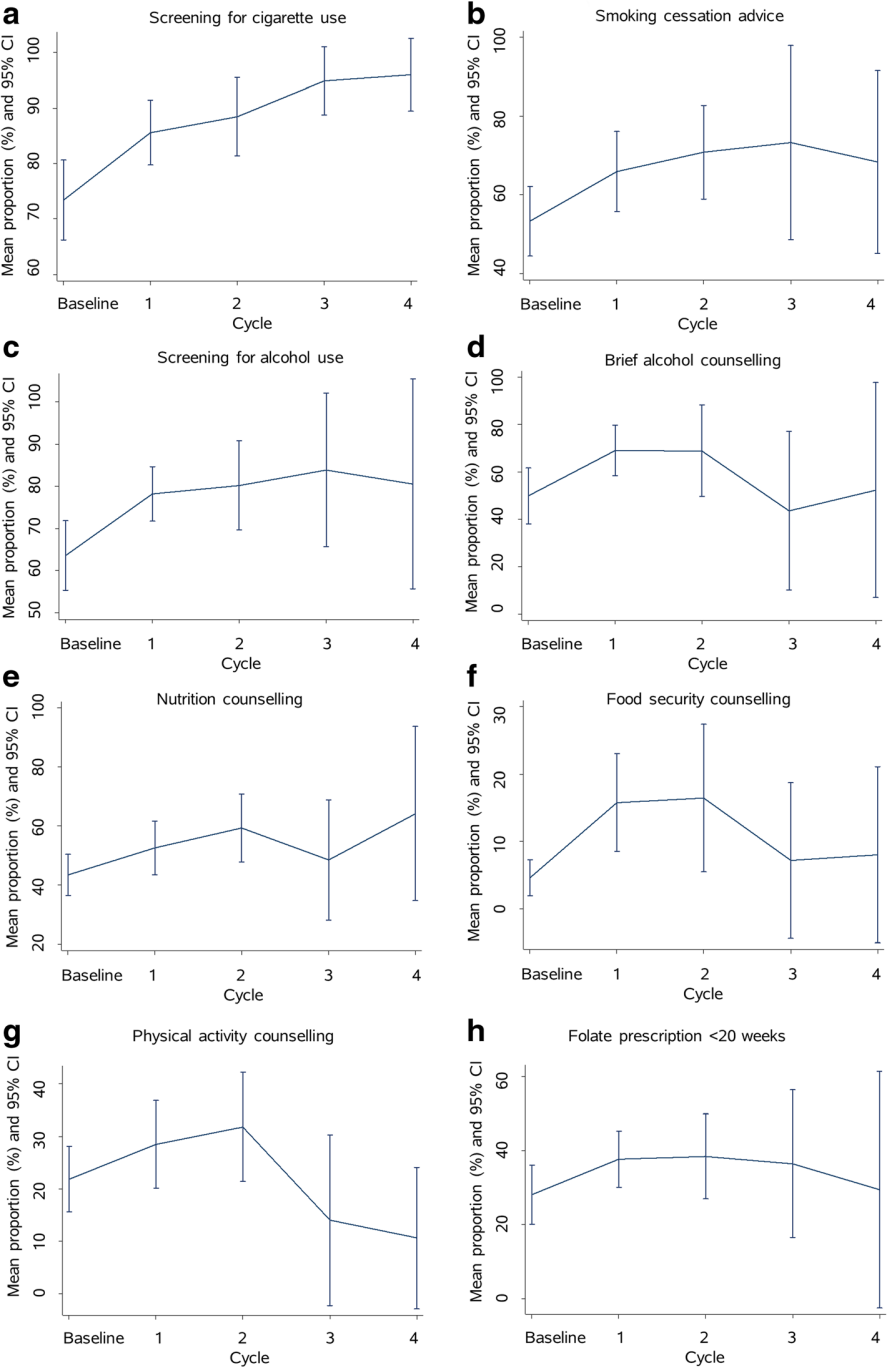
Mean proportion (and 95 % confidence interval) of women per health center receiving each pregnancy care measure after each cycle (longitudinal data)

Figure 1 shows the average provision of each outcome measure after each cycle. This indicates that the average proportions of women receiving screening for cigarette use, cigarette cessation advice, screening for alcohol use and nutrition counselling were greater after completion of one or more CQI cycles and that some improvements were maintained over a number of cycles.

### Associations between SAT scores and pregnancy care measures (PHC level)

Univariable linear regression analysis investigated whether PHCs with better organizational systems had greater provision of outcome measures. SAT data were available for 27 PHCs (54 %) and 21 PHCs had SAT data for more than one CQI cycle. Associations between SAT scores and food security counselling or physical activity were not assessed due to low provision. There were statistically significant positive associations between self-ratings of organizational systems and all outcome measures except screening for cigarette use and brief alcohol counselling (Table 3). For example, for every one-unit increase in overall SAT score, the average proportion of women screened for alcohol use increased by 6.8 % (*p* = 0.04). No statistically significant associations were observed between changes in SAT scores and changes in any outcome measure (all *p*-values ≥0.05, data not shown).

### Associations between PHC characteristics and pregnancy care measures

Chi-square tests investigated associations between PHC characteristics and provision of key outcome measures relating to smoking, alcohol, nutrition and early pregnancy folate prescription (food security counselling and physical activity were not assessed due to low provision). More government-operated PHCs than community-controlled PHCs were among those PHCs with the highest provision of brief alcohol counselling (45 and 12 % *p* = 0.02). When changes in provision were investigated, we found that more community-controlled PHCs than government-operated PHCs were among those PHCs with the most improvement in brief alcohol counselling (53 and 21 % *p* = 0.03). More city/regional PHCs than remote PHCs were among those with most improvement in brief alcohol counselling (67 and 25 % *p* = 0.04). More PHCs in Western Australia than other states were among those with most improvement in early pregnancy folate prescription (83 and 0-38 % *p* = 0.03). No other statistically significant results were observed (*p*-values ≥0.05, data not shown).

## Discussion

This large, longitudinal study supports the use of CQI activities to improve provision of recommended pregnancy care for Indigenous women attending PHCs. It also provides evidence of the potential for quality improvement efforts targeting underlying systemic issues to improve pregnancy care in primary care settings in general. Women were more likely to have documented provision of screening and counselling regarding lifestyle factors if they attended after PHCs completed one or more CQI cycles. Opportunities for further improvement included lifestyle counselling and folate prescription. Better self-rated organizational systems were associated with greater provision of outcome measures, and PHC governance and location were associated with pregnancy care provision and changes in service delivery.

Greater provision of pregnancy care regarding lifestyle-related risk factors was associated with participation in this CQI initiative and supports the incorporation of CQI activities into primary care settings to improve pregnancy care. This is an important contribution as there is relatively little international research regarding CQI in pregnancy care and most has been in hospital settings [24–27]. Additionally, as the ABCD Partnership involves multiple PHCs, it is uniquely placed to identify systemic issues that affect performance across a wide range of settings rather than providing information only relevant locally. Collaborative, multi-center, hospital-based CQI networks in the United States of America (USA) have recently reported decreases in scheduled deliveries without a medical indication (between 36–39 weeks gestation), decreases in bloodstream infections in preterm infants, and improvements in processes to support breastfeeding [24, 25]. CQI approaches have been used in single-center, hospital-based settings to improve care for diabetes in pregnancy in the USA and to improve coordination of antenatal and postnatal care in England [26, 27]. In the Australian context, the national pregnancy care guidelines recommend enquiry about lifestyle-related risk factors for adverse pregnancy outcomes to enable explanation of these risks, offer appropriate lifestyle counselling and arrange treatment or referral if required [8]. Increased provision of these components may contribute to improving the health of Indigenous mothers, closing the gap in perinatal outcomes between Indigenous and other Australian women, and potentially improving the health of the children long-term through an improved intra-uterine environment. Some of the observed increases may reflect improved documentation of existing provision rather than changes in provision, but accurate documentation itself is an important quality indicator, as it improves continuity of care and increases efficiency. This study also identified lifestyle counselling and folate prescription as specific areas where further improvements are needed. Barriers to peri-conception folate prescription and use should be investigated to identify how health center systems and processes could be improved to support this. Prevention of smoking in pregnancy has been described as the single intervention with the biggest potential impact on perinatal outcomes in Indigenous populations in Australia [28]. The findings presented here show that, while enquiry about smoking was almost universal by cycle four, more than one quarter of women who smoked were not provided with cessation advice. Further research is needed to identify optimal ways to support Indigenous women to implement smoking cessation and prevention strategies. To further facilitate benchmarking and implementation of best practice, an important part of the ABCD Research Partnership’s approach is providing these aggregated, large-scale findings to stakeholders at regular meetings designed to share knowledge and focus research priorities [20]. Wider engagement of stakeholders is supported through dissemination of draft reports and encouraging participation in interpreting and using findings to improve practice and policy. An example of such a multidisciplinary strategy is the recently developed health promotion CQI tool [29].

Consistent with the systems focus of CQI, the observed greater likelihood of receiving screening and counselling was not confined to an isolated area but observed across all measures investigated. Better self-rated organizational systems were associated with greater provision of outcomes measures and, in particular, information systems/decision support, and organizational influence/integration were associated with greater provision of several measures, suggesting they affect performance across multiple settings. These may be areas for targeted systems level improvement efforts to improve pregnancy care generally. These findings are supported by related work within the ABCD Research Partnership that noted positive associations between higher SAT scores and higher provision of pregnancy care measures [30]. This is also consistent with diabetes care research, in which greater SAT subscale scores were associated with better adherence to delivery of recommended services [22]. Together these findings indicate that the state of development of organizational systems is reflected in quality of care and highlight the importance of focusing on improving systems and processes, rather than local, isolated problems. However, in the current research, no statistically significant association was observed between changes in SAT scores and changes in provision of outcome measures, potentially reflecting the small number of PHCs for which SAT data were available.

Recent Australian research has emphasized the importance of providing pregnancy care in a culturally safe environment [31–34]. The current research focused on the overarching strategy of strengthening organizational systems and processes to improve quality of care. Within this, PHCs may have focused their CQI activities on provision of culturally appropriate care or on other aspects of organizational systems. Other research using a quality improvement framework reported improvements in pregnancy care provision at an urban community-controlled health service compared to a historical cohort [35]. Our findings are consistent with this and further indicate CQI’s large-scale potential within primary care by including many PHCs across multiple jurisdictions, serving small and large communities, in urban and remote settings.

Better adherence to recommended service delivery also appeared to be sustained with continuing PHC involvement over a number of cycles, with some evidence that further improvements were gained from each additional CQI cycle. This highlights the importance of supporting long-term participation in CQI, consistent with previous reports of improvements in pregnancy care over six years of annual clinical audits (2000–2005) [35]. The research presented here also provides important long-term information about current provision of pregnancy care by PHCs in Indigenous communities. While some research has recently evaluated pregnancy care for Indigenous women over several years [31–33], these programs were implemented at one site or in one geographic region, limiting generalizability of the findings.

Our findings indicate that PHC characteristics and context may influence pregnancy care provision and changes in service delivery, however there was no indication that the CQI initiative was only successful in one specific setting. Previous research has identified that maternal risk factors, maternal health service delivery and pregnancy outcomes vary between different settings, such as geographical region and remoteness [18, 36]. Given this diversity, there are likely to be benefits from identifying programs that can be successfully implemented across a range of settings. A strength of collaborative, system-based, CQI initiatives and research is the ability to draw on a shared understanding of issues encountered across settings as well as specific experiences or insights of PHCs [20].

### Limitations

One limitation of this research is that systems assessment data were available for select PHCs, which reduced the sample size and therefore the statistical power to detect small effects. While PHC was included as a random effect in the individual-level regression analysis, sample size did not permit adjustment for confounders in the health center level regression analysis. Selection bias is also possible as this study only included the subset of PHCs participating in the One21seventy initiative that consented to make their data available for research purposes. Also, the data may not be representative of PHCs not participating in the CQI initiative, but there are currently no comparable sources of data in Australian primary care to form a comparison group. As most PHCs were in remote areas and the PHCs that had completed more cycles were mainly community-governed, the findings may not be generalizable beyond these settings. Few PHCs had completed three or four CQI cycles, so the numbers of women and babies were too small to permit meaningful comparisons of birth outcomes. Additionally, further evaluation of the different ways that brief counselling may be delivered was not possible. Despite these limitations, this is the largest and most comprehensive quality improvement project involving PHCs, serving predominantly Indigenous populations, participating in repeated CQI cycles over a number of years. The relevance to primary care in general is improved by inclusion of PHCs across a range of settings.

## Conclusion

Women attending PHCs that had participated in continuous quality improvement activities were more likely to receive recommended pregnancy care related to screening and brief interventions for modifiable lifestyle-related risk factors for key adverse pregnancy outcomes. These findings support incorporation of continuous quality improvement activities, addressing underlying systemic issues, into primary care settings to improve pregnancy care. Clear areas were identified where further improvements in provision of care are needed to improve pregnancy outcomes in Aboriginal and Torres Strait Islander women in Australia.

### Ethics and consent to participate

The study was approved by human research ethics committees in the Northern Territory, New South Wales, Western Australia and Queensland, and by their Indigenous sub-committees where required [18]. The de-identified data analyses presented here were approved by the Monash University Human Research Ethics Committee (CF12/3434-2012001670). Formal, written agreement was provided by each health center to participate in the ABCD National Research Partnership, including the analysis of data for research purposes [19]. Strict privacy and confidentiality protocols protected the privacy of clients and the identity of participating health centers [19]. All regional research ethics committees accepted that obtaining individual client consent to audit health records would be impractical [19].

## Availability of data and materials

Enquiries regarding the datasets and materials supporting these findings should be addressed to the ABCD National Research Partnership Committee, care of Professor Ross Bailie: ross.bailie@menzies.edu.au.

## Abbreviations

ABCD: audit and best practice for chronic disease national research partnership
CQI: continuous quality improvement
PHC: primary health care center
SAT: systems assessment tool
